# Smoothed particle hydrodynamics based FSI simulation of the native and mechanical heart valves in a patient-specific aortic model

**DOI:** 10.1038/s41598-024-57177-w

**Published:** 2024-03-21

**Authors:** Sumanta Laha, Georgios Fourtakas, Prasanta K. Das, Amir Keshmiri

**Affiliations:** 1 School of Engineering, University of Manchester, Manchester, M13 9PL UK; 2 Department of Mechanical Engineering, IIT Kharagpur, Kharagpur, 721302 India; 3 Manchester University NHS Foundation Trust, Manchester, M13 9PL UK

**Keywords:** Biomedical engineering, Computational models

## Abstract

The failure of the aortic heart valve is common, resulting in deterioration of the pumping function of the heart. For the end stage valve failure, bi-leaflet mechanical valve (most popular artificial valve) is implanted. However, due to its non-physiological behaviour, a significant alteration is observed in the normal haemodynamics of the aorta. While in-vivo experimentation of a human heart valve (native and artificial) is a formidable task, in-silico study using computational fluid dynamics (CFD) with fluid structure interaction (FSI) is an effective and economic tool for investigating the haemodynamics of natural and artificial heart valves. In the present work, a haemodynamic model of a natural and mechanical heart valve has been developed using meshless particle-based smoothed particle hydrodynamics (SPH). In order to further enhance its clinical relevance, this study employs a patient-specific vascular geometry and presents a successful validation against traditional finite volume method and 4D magnetic resonance imaging (MRI) data. The results have demonstrated that SPH is ideally suited to simulate the heart valve function due to its Lagrangian description of motion, which is a favourable feature for FSI. In addition, a novel methodology for the estimation of the wall shear stress (WSS) and other related haemodynamic parameters have been proposed from the SPH perspective. Finally, a detailed comparison of the haemodynamic parameters has been carried out for both native and mechanical aortic valve, with a particular emphasis on the clinical risks associated with the mechanical valve.

## Introduction

Heart valves are crucial components of human hearts which ensure the proper directions of systemic and pulmonary circulation and unidirectional blood flow through the different chambers of the heart. There are four valves in the heart—the aortic valve, the mitral valve, the tricuspid valve, and the pulmonary valve—each with its own specific function. For a healthy human, the valve needs to operate approximately 100,000 times in a day. The occurrence of heart valve failures is a common and that leads to serious health hazards^1^. Though the general causes of failure of the heart valves could be congenital defects, infections, or degenerative conditions, the failure rate of the aortic valve is much higher compared to its counterparts. When the heart valve fails, it can cause a range of symptoms including shortness of breath, chest pain, and fatigue. If left untreated, heart valve failure can lead to more serious complications such as heart failure, stroke, or even death. Although medicinal treatment may be effective for initial stages of valvular diseases, implantation of a prosthetic valve is necessary for advanced cases where the native heart valves have failed completely. The most used prosthetic valve is the bi-leaflet mechanical heart valve (BMHV) which exhibits non-physiological fluid behaviour in the vasculature and that certainly introduces prosthetic induced risk and thrombosis.

Computational fluid dynamics (CFD) can play an important role in exploring the operation of both native and artificial heart valves under normal and malfunction conditions. As the heart valve operates with constant and swift motion, it is essential to integrate a FSI approach into the CFD model. This ensures an accurate representation of the valve's dynamic movement. The technique can be used profitably to predict the prosthetic induced risks and finally to improve the design of the mechanical valves^2–5^. The present study aims to demonstrate a comprehensive comparative analysis between the haemodynamic behaviour of native and mechanical aortic valve. In order to do that a detailed FSI model of both flexible native and rigid mechanical aortic heart valve has been carried out.

Numerous efforts have been made to model the bi-leaflet mechanical heart valve (BMHV) through CFD modelling^6–12^. In most of the studies, an idealised geometry of the BMHV and an anatomically reasonable geometry of the aorta have been used for the analysis with conventional finite volume method (FVM). Dasi et al.^13^ have conducted a rigorous study for understanding the vorticity dynamics of the bi-leaflet heart valves downstream of the valve. In order to model heart valve haemodynamics more rigorously, fluid–structure interaction (FSI) techniques have also been employed. In this method, the CFD model is coupled with a structural model of the valves. Researchers have also adopted this method to simulate the opening and closing behaviour of the valve leaflets^14–16^. Bongert et al.^6^ developed a 3D CFD model of the BMHV (aortic valve) with patient specific vascular geometry taken from 4D magnetic resonance imaging (MRI) data. They carried out several simulations to find the optimal mounting angle of the BMHV in the aortic root of any particular patient.

Previous research has also shown that modelling mechanical heart valves, specifically the bi-leaflet heart valve, is relatively easier compared to modelling flexible native valves^17^ due to the rigid structure of the BMHV. Yan et al.^18^ developed an FSI model of a native aortic valve using immersed boundary method. LS-DYNA (LSTC, Livermore, CA, United States and ANSYS Inc., Canonsburg, PA, United States) were used to implement the FSI processes in which, a detailed comparison of the bicuspid native aortic valve against the general tricuspid native aortic heart valve was conducted to understand the risk associated with the bicuspid valve phenotypes. Mutlu et al.^19^ investigated the fluid dynamics abnormalities of a calcified aortic valve by using Lagrangian coherent structures coupled Eulerian approach. Patient specific sizing was chosen for modelling the aortic sinus and valve geometry, while spring-based mechanism and remeshing algorithms of ANSYS Workbench 19.2 (Canonsburg, PA, USA) was used to enable FSI modelling. ANSYS Fluent was employed to create a model of the fluid domain in order to analyse the flow velocity and pressure distribution. Simultaneously, the ANSYS transient structural module was utilised to simulate the deformations of the leaflet within the solid domain. Spühler et al.^20^ presented a new methodology for modelling native heart valves, which employs a unified continuum arbitrary Lagrangian–Eulerian (ALE) finite element method (FEM) coupled model. While the proposed model is capable of accurately simulating the flexible aortic valve, meshing and running FSI is a challenging task. Discretising such a coupled problem is complex and requires additional algorithms for mesh smoothing/remeshing to maintain numerical accuracy and robustness.

From the above literature review, it can be observed that a challenge on the above studies is the FSI due to the motion of the valve leaflets and dynamic mesh generation. Different techniques such as ALE^21^ and Immersed Boundary Method (IBM)^22^ have been adopted to address this issue. However, the major limitation of the ALE is mesh quality and equality which may be disturbed with the larger deformation due to FSI. The continuous remeshing of the ALE also augment the computational cost and complicacy. On the other hand, Abbas et al.^17^ clearly demonstrated the effectiveness and advantages of the mesh free FSI methods such as the lattice-Boltzmann method (LBM) and SPH for the heart valves simulations.

In recent times, numerous efforts have been devoted to model general cardiovascular FSI problems using the meshless Smoothed Particle Hydrodynamics (SPH) approach. Faiz et al.^23^ extensively documented the various applications of SPH in cardiovascular fluid mechanics and also elucidated the simple implementation of FSI for these issues compared to traditional FVM approaches. Lluch et al.^24^ exemplified the advantages of the SPH method in cardiovascular problems, highlighting its ability to eliminate the need for mesh and consequently mitigate errors related to mesh deformation. This emerging trend underscores the significance and imperative for developing a robust SPH-based model tailored for intricate cardiovascular simulations. Furthermore, Wenbin et al.^25^ conducted a thorough fluid–structure interaction study on the aortic and mitral valves, employing a real left ventricular model. They coupled a SPH model with a nonlinear finite element (FE) model. The FE model simulated the wall movement of the left ventricle and the dynamics of the valve leaflet throughout the entire cardiac cycle. Their findings revealed that the SPH-coupled FSI model provided more accurate and realistic valve dynamics and deformation compared to a single finite element model. Dabiri et al.^26^ explored the impact of MitraClip (MC) implantation on mitigating complexities associated with tricuspid valve regurgitation using a fluid–structure interaction model. They used SPH to simulate the effects of MC location on tricuspid valve closure and regurgitation within the commercially available Abaqus 614.3 software platform.

In addition, Monteleone et al.^27^ introduced a novel monophysics approach for simulating cardiovascular fluid–structure interaction through the use of the SPH method. They emphasized the critical need for particle-based methods such as SPH in cardiovascular FSI, especially in simulating flexible objects. The method was validated through an immersed beam test case, and the results are promising for adopting the proposed model in cardiovascular FSI problems.

Nasar et al.^28^ developed a novel method to model the fluid interactions with the slender flexible model by using Vector-based discrete element method based Eulerian SPH. Furthermore, they have investigated some of the simple 2D test cases of the healthy venous valves in the human cardiovascular system. While some other efforts are also given to model the heart valve by the mesh free FSI approach, the number is limited^29^. This certainly brings out a significant research gap in the field of heart valve modelling using mesh-free FSI techniques particularly by SPH. A companion paper from the same authors has pioneered in this field^30^, to which the reader is referred, to see the initial modelling of the MHV by SPH techniques in more detail.

The meshless nature of the particle-based method such as SPH enables this approach to handle complex geometries and deformations more easily than mesh-based method like ALE or IBM resulting the easy implementation of the complex FSI. Additionally, SPH handles the interfaces between blood, artery and valve walls naturally, reducing the need for the explicit interface-tracking methods required by IBM and ALE. This particular advantage makes it easier to describe complex fluid–structure interactions in cardiovascular simulations since it is possible to accurately capture the interactions between blood and artery walls. Moreover, being a Lagrangian scheme, time history of the flow is readily available in the SPH simulation while ALE and IBM combine both Lagrangian and Eulerian frameworks and may encounter difficulties in accurately representing individual fluid behaviour.

As a significant step to fill the mentioned research gap^17^, in the present study, a SPH based FSI model has been proposed for the simulation of heart valves. For simulation of the artificial valve a 25 mm St. Jude bi-leaflet mechanical heart valve has been considered inside a patient specific aortic geometry. For comparison the operation of a native valve in the same geometry has also been investigated. To the best of our knowledge, the present study pioneers the modelling of the flexible valve in a patient specific aortic vasculature through SPH. In addition, a novel methodology has been proposed for estimating the wall shear stress and other important haemodynamic matrices such as oscillatory shear index (OSI), endothelial cell activation potential (ECAP) extending the scope of SPH. Finally, the present study focuses on a detailed comparison of performance of both the valves based on the haemodynamic parameters and effective orifice area (EOA). This comparison clearly pinpoints the regions subjected to the prosthetic induced thrombosis.

## Methods

### Smoothed particle hydrodynamics

The governing equations of hydrodynamics problems comprises of partial differential equations (PDEs) of field variables, namely the mass and momentum conservation equations. In order to solve the above-mentioned equations, the domain needs to be discretise and generate an approximate solution of the field functions at discrete locations.

In the SPH method, the continuous approximation of a scalar field function $A\left( r \right)$ at a point, *r* in a 1-D domain takes an integral representation form and can be written as,

1 $$A(r): = \int {W(r - r^{\prime},\;h)A(r^{\prime})\; d\omega }$$

where *W* is a smoothing function or smoothing kernel, *h* is the smoothing length in the support domain of the kernel function and *ω* is the volume of the integral.

The discrete form of Eq. (1) can be written as,

2 $$A_{i} = \mathop \sum \limits_{j}^{N} \;W\;(r_{i} - r_{j} ,\;h)A_{j} V_{j}$$

where *ω* is discretised in *N* computational nodes or particles. Herein, *i* and *j* denote the interpolating and neighbouring particles respectively, and *V* is the volume of the particle such as $V = m /\rho$ with *m* the mass and *ρ* the density of the particle.

Choosing an appropriate smoothing function is crucial for getting a proper solution. Several smoothing kernels are reported in the literature^31^. In the present work, the Wendland *C*_2_ kernel has been chosen as it is a higher-order (5th) kernel and hence, it can capture higher-order effects with its improved accuracy^31^. The Wendland kernel reads as follows^32^,

3 $$W(r,h) = \left\{ {\begin{array}{*{20}l} {\alpha_{d} \left( {1 - \frac{q}{2}} \right)^{4} } & {\left( {2q + 1} \right) ,} & {\quad 0 \le q \le 2} \\ {} & 0 & {} \\ 0 & {} & {\quad q > 2} \\ \end{array} } \right\}$$

where $$q = {\raise0.7ex\hbox{${\left| {r_{ij} } \right|}$} \!\mathord{\left/ {\vphantom {{\left| {r_{ij} } \right|} h}}\right.\kern-0pt} \!\lower0.7ex\hbox{$h$}}$$ and $$\alpha_{d}$$ is $$\frac{3}{4h},\;\frac{7}{{4\pi h^{2} }}, \;\frac{21}{{16\pi h^{3} }}$$ for the 1D, 2D, and 3D respectively. For further information on the SPH method the reader is directed to^33^ and^34^.

### Governing equations for hydrodynamics

#### Continuity equation

The majority of the hydrodynamic problems are solved by the weakly compressible SPH (WCSPH) scheme. Throughout the SPH simulation, the mass of each particle is kept constant while the density of the particles changes according to the continuity equation. The general form of the continuity equation is as follows^35^,

4 $$\frac{D\rho }{{Dt}} = - \rho {\mathbf{\nabla }} \cdot ({\mathbf{u}})$$

whereas the SPH discrete form of Eq. (4) can takes the following form,

5 $$\frac{{{\text{D}}\rho }}{{{\text{Dt}}}} = \rho_{{\text{i}}} \mathop \sum \limits_{{{\text{j}} = 1}}^{{\text{N}}} \frac{{{\text{m}}_{{\text{j}}} }}{{\rho_{{\text{j}}} }}({\mathbf{u}}_{{\text{i}}} - {\mathbf{u}}_{{\text{j}}} ) \cdot \left( {{\mathbf{\nabla }}{\text{W}}_{{{\text{ij}}}} } \right)$$

where **u** is the velocity of the particle and *t* denotes physical time.

#### Momentum equation

The momentum conservation equation can be written as,

6 $$\frac{{{\text{D}}{\mathbf{u}}}}{Dt} = - \frac{1}{\rho }{\mathbf{\nabla }}p + \Gamma + {\mathbf{f}}$$

where $p$ is the pressure Γ is the dissipative term and **f** represents the body force term. In SPH notation the above Eq. (6) can be expressed as,

7 $$\frac{{{\text{D}}{\mathbf{u}}}}{{{\text{Dt}}}} = - \mathop \sum \limits_{{{\text{j}} = 1}}^{{\text{N}}} \left( {\frac{{p_{i} + p_{j} }}{{\rho_{i} \rho_{i} }}} \right)\left( {{\mathbf{\nabla }}{\text{W}}_{{{\text{ij}}}} } \right){\text{m}}_{{\text{j}}} + \left\langle \Gamma \right\rangle + {\mathbf{f}}{ }$$

The above equation satisfies the momentum conservation. The dissipative term has been calculated by using Sub-Particle Scale (SPS) turbulence which is a combination of Shao and Lo operator^36^. The detailed description of the model and other governing equations has been discussed in the supplementary document.

### Model description and boundary conditions

The current research presents a numerical model that utilises a Lagrangian mesh-free WCSPH approach to study the hydrodynamics of a realistic bi-leaflet mechanical aortic heart valve. The present 3D unsteady, turbulent, FSI model enables the investigation of various haemodynamic parameters, including velocity, vorticity, pressure, Wall Shear Stress (WSS) and WSS-based metrics in the flow domain. The present approach has been applied successfully to other cardiovascular flow problems in the past^4,37–39^.

**Figure 1:**
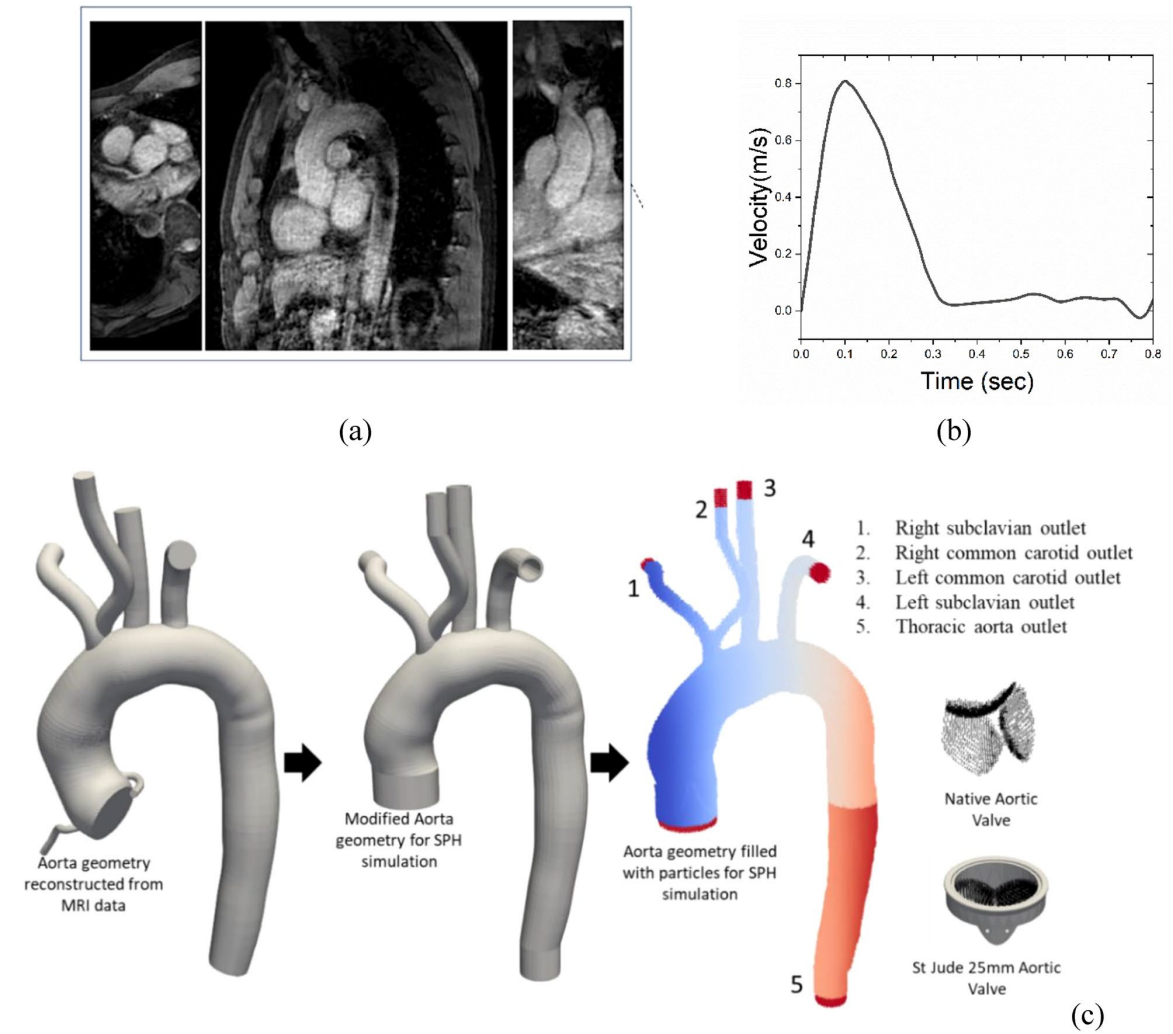
Details of the present. (**a**) DICOM images of the MRI; (**b**) inlet velocity profile; (**c**) reconstructed CAD model of the aorta from MRI data and schematic of the valves^40^.

In order to augment the clinical significance, realistic material properties of the valve have been employed and the patient specific aortic geometry, reconstructed from 4D MRI data has been chosen for the simulation^40^. In the present model the aortic wall is represented as a rigid structure, while the leaflet of the native valves is modelled as a deformable (elastic) object. The mechanical properties of the healthy aortic valves are derived from literature sources, with the Young modulus and the Poisson ratio set at 1 MPa and 0.49, respectively^41^. The density of the leaflet is chosen^19,42^ as 1100 kg/m^3^. The details of the present model have been shown in Fig. 1. The reconstructed aorta geometry has one inlet from the left ventricular side and five outlets such as thoracic aorta outlet (5), right subclavian outlet (1), right common carotid outlet (2), left common carotid outlet (3) and left subclavian outlet (4). It may be noted that the actual reconstructed model has been modified marginally for implanting inlet/outlet condition in the SPH solver.

It is important to note that in the present model the smoothing length is chosen in such a way that every interpolating particle receives sufficient kernel support from its neighbouring particles. The smoothing length for both the structure and fluid is set at 1.4 times the particle spacing. This study employed DualSPHysics^43^, an SPH open-source C++-based code that utilises graphics processing units (GPUs) for hardware acceleration. The code utilises the WCSPH formulation discussed in methods section. To accelerate the simulations, an NVIDIA RTX A1000 GPU card was used were the present simulations with 900 K particles completed in 18 h. The construction of the leaflets for both the flexible and rigid mechanical valves involves particles arranged in three layers.

### FSI coupling for the mechanical and the native valve

To explore the interaction between mechanical valve movement and the flow field, the project Chrono^44^, implemented in DualSPHysics^45,46^, has been utilised. Project Chrono is a physics-based modelling and simulation infrastructure with an open-source design implemented in C++. In the initial step, DualSPHysics computes the forces acting on the fluid-driven object, and then the linear and rotational acceleration is applied to the Chrono object (at the centre of mass). Subsequently, Chrono calculates the movement of the object and conveys information about the new position, velocities, and the adjusted centre of mass back to DualSPHysics. Within Chrono, rigid bodies are considered as a subset of the SPH particles, and variables associated with these particles have been integrated with time. Newton's equations for rigid bodies are employed in the computation^45^. The discretized equations are outlined as follows,

8 $$M\frac{{d{\mathbf{V}}}}{dt} = \mathop \sum \limits_{k \in I} { }m_{k} {\mathbf{f}}_{k}$$

9 $${\mathbf{I}}\frac{{d{{\varvec{\Omega}}}}}{dt} = \mathop \sum \limits_{k \in I} { }m_{k} \left( {{\mathbf{r}}_{k} - {\mathbf{R}}_{0} } \right) \times {\mathbf{f}}_{k}$$

where *M* is the mass of the rigid body, *V* is the velocity, *I* is the inertial tensor, *R*_0_ is the center of mass and $\Omega$ is the angular velocity of the body I. $f_{k}$ is force per unit mass applied to the particle *k* of the rigid body *I*, which is the combined force of the fluid resultant to the boundary particle. The interactions between the fluid–fluid particle and fluid–solid boundary particle have been calculated based on Eq. (7). Thereafter, the Chrono-Engine computes the linear and angular velocities and the centre of mass for the rigid body^44^ based on the given physical constrains. In turn, DualSPHysics computes the velocity for each boundary particle according to,

10 $${\mathbf{u}}_{k} = {\mathbf{V}} + {{\varvec{\Omega}}} \times \left( {{\mathbf{r}}_{k} - {\mathbf{R}}_{0} } \right)$$

updating the system for fluid and boundary particles^46^. To elaborate further on the calculation process and implementation details, readers are referred to^43,45^ for a more comprehensive discussion. Furthermore, the detailed description of the mechanical valve modelling by using Chrono could be found at our previous work^30^.

In order to model the FSI of the flexible native heart valve, the method proposed by O’Connor et al.^47^ has been utilised, along with its implementation in the DualSPHysics. Here, the movement of the boundary particles (flexible) must be considered based on the stress–strain tensor unlike the rigid body dynamics where only the movement of the centre of mass is considered. In the primary step the momentum equation for the flexible structure is solved which reads as

11 $$\rho J = \rho_{0}$$

12 $$\frac{dv}{{dt}} = \frac{1}{{\rho_{0} }}\nabla_{0} \cdot {\mathbf{P}} + {\mathbf{g}},$$

where J is the Jacobian determinant of the deformation gradient, and P is the first Piola–Kirchhoff (PK1) stress tensor. Here the subscript zero denotes the operator is subjected to the material reference frame. In order to calculate the stress, deformation gradient must be computed. The detailed step by step procedure for the implementation of the said FSI technique can be found at the previous work done by O’Connor et al.^47^.

In the present work, while using DualSPHysics^43^, the Dynamic Boundary Condition (DBC)^43^ has been used instead of the Modified DBC (mDBC)^48^. Though the later provides a better implementation of no slip condition, it poses a greater challenge in the present study for generating the boundary normals for complex geometries. However, the emphasis of the present investigation is the implementation of SPH based FSI for intricate problems like heart valve dynamics. Despite the use of DBC, the finely calibrated particle resolution allows us to reasonably estimate near-wall functions; however, achieving a proper no-slip condition at the wall remains a challenging endeavour and leaves a scope for further development.

In the present work, two separate cases studies have been proposed. In the first case, a geometry similar to the native aortic valve was placed within the patient's specific aorta. In the second case, the geometry of a 25 mm St Jude bi-leaflet mechanical valve (manufactured by St. Jude Medical, Inc, USA) was incorporated in the model.

Physiologically reasonable pulsatile velocity profile has been imposed from the inlet side of the geometry, while the outlets are prescribed with a constant pressure. In the SPH method, an inlet/outlet boundary^43^ has been defined as a buffer layer consisting of buffer fluid particles at the inlet/outlet region. Buffer zones are implemented to reduce inaccuracies that occur near boundaries due to kernel truncation, and their purpose is to rectify the creation or removal of particles within these regions to prevent voids. To maintain flow conditions consistent with the boundaries, velocities and/or pressures can be assigned to particles within the buffer region. The algorithm is intentionally constructed to enable the extrapolation of physical properties for these buffer particles from the fluid domain, employing a first-order consistent approach that relies on ghost points strategically placed within the fluid domain near the boundary. For further information on the algorithm, readers can refer to the details provided in the work of Tafuni et al.^49^. In the present study 5-layer thick buffer zone has been created to ensure complete kernel support at all flow conditions. At the inlet, any fluid particle that enters the buffer zone is removed from the simulation, while at the outlet, fluid particles that enter the buffer zone are converted into buffer particles. The initial density and viscosity of the blood were set to 1060 kg/m^3^ and 0.003 Pa·s (or 3cP), respectively and the blood is assumed to be a Newtonian fluid^50–52^. To observe the leaflet’s behaviour during the opening and closing cycle, simulations were conducted for a real-time duration of two cardiac cycle (0.8 s each). Figure 1 shows the schematic representation of the patient specific aorta and the native/mechanical heart valve. The velocity profile exhibits its peak systole at 0.1 s with a maximum Reynolds number of 7000.

### Formulation of the hemodynamic metrics

#### Wall shear stress (WSS)

**Figure 2:**
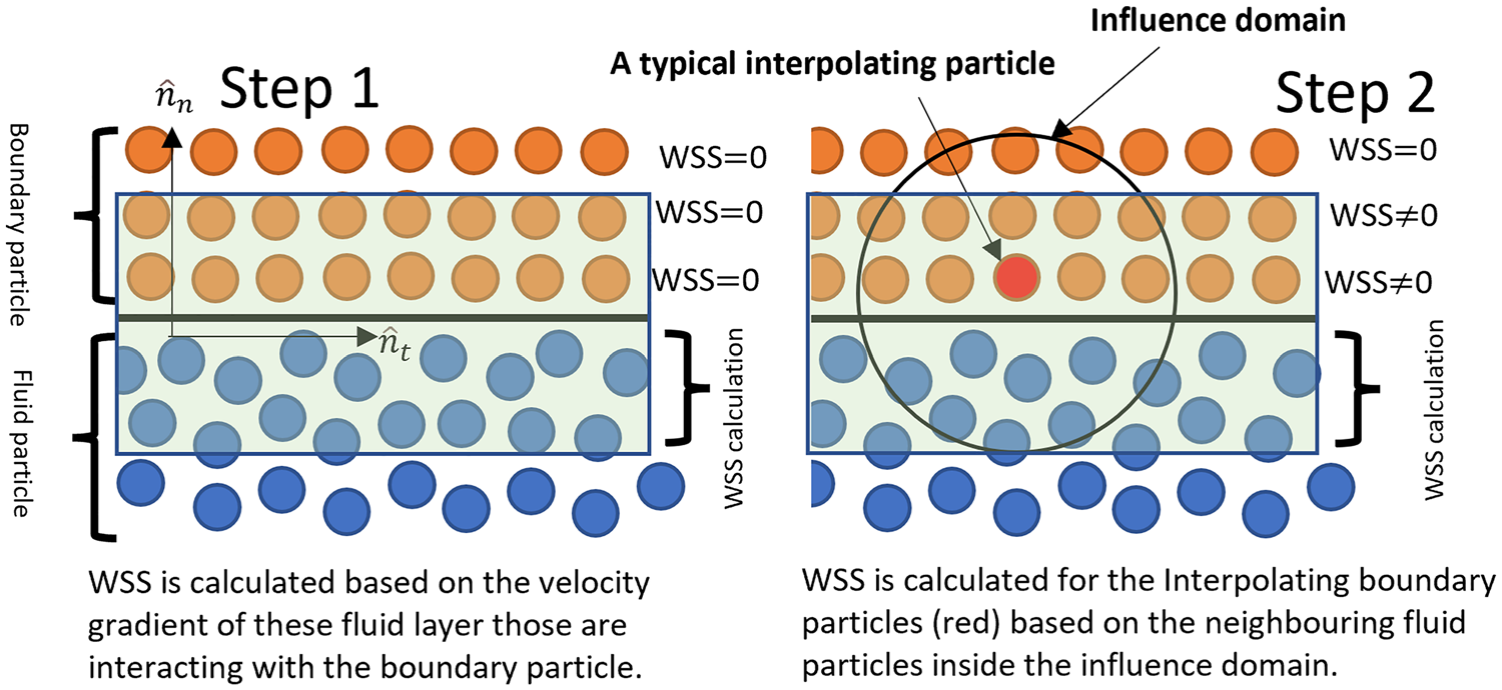
Schematic diagram of the WSS estimation on the wall.

The wall shear stress in the aorta is an important haemodynamic parameter which has successfully been used by numerous researchers in the past^40,52–54^. In general, WSS must be calculated at the wall surface. However, as mentioned earlier, DBC boundary condition has been employed in the present work. Under the DBC, the velocity of the boundary particles is assumed to be zero, leading to an inconsistent no-slip wall boundary condition which influences the accuracy of the WSS calculation. Therefore, one of the contributions of the present work was to implement a method in the code to calculate the approximated WSS at the last fluid layer adjacent to the wall surface. Finally, the shear stress has been calculated at the wall as elaborated in the supplementary material. The schematic of the WSS estimation is explained in Fig. 2. The sets of fluid and boundary particles participating in the WSS calculation is shown in a shaded region. For a laminar flow, the wall shear stress can be formulated as,

13 $$WSS = 2\mu S_{ij} \hat{n}_{t}$$

14 $$S_{ij} { } = \frac{1}{2}\left[ {\nabla {\varvec{u}} + \nabla {\varvec{u}}^{T} } \right]$$

and for the turbulent flow, Smagorinsky Large Eddy Simulation (LES) model has been utilised in which the stress tensor can be read as^55^,

15 $$\tau_{i,j} = \mu_{T} \left( {2S_{ij} - \frac{2}{3}k\delta_{ij} - \frac{2}{3}C_{I} k\delta_{ij} \Delta^{2} \delta_{ij} \left| {S_{ij}^{2} } \right|} \right)$$

16 $$\mu_{T} = \rho \left( {C_{s} \Delta l} \right)^{2} \sqrt {2S_{ij} S_{ij} }$$

17 $$WSS = \tau_{i,j} \hat{n}_{t}$$

where, $\mu$ and $\mu_{T}$ are the laminar and turbulent dynamics viscosity, respectively, $\Delta l$ is the smallest length scale and $C_{s}$ is the Smagorinsky constant. ${ }S_{ij} { }$ is an element of the SPS strain tensor. $C_{I} ,k$ are the constant value and $\delta_{ij}$ is the Kronecker delta. $\hat{n}_{t}$ is the tangent of the respective direction^55^. Further discussion of the WSS estimations could be found at the supplementary document.

#### Time averaged wall shear stress (TAWSS)

In the cardiovascular simulation, the absolute WSS on its own, cannot accurately represent the entire haemodynamic effect of a full cardiac cycle. A time averaged wall shear stress (TAWSS) over an entire cardiac cycle is an effective way to estimate the cumulative haemodynamic effect and can be calculated as follows,

18 $${\text{TAWSS}} = \frac{1}{T}\mathop \int \nolimits_{0}^{T} \left| {\tau_{wall} } \right|dt$$

#### Oscillatory shear index (OSI)

The oscillatory shear index (OSI) is another parameter used to quantify the directionality and magnitude of shear stress changes during the cardiac cycle in arterial blood flow. The OSI has been identified as one of the important markers for detecting the atheroprone regions of the vasculature and can be formulated as^56^,

19 $${\text{OSI }} = \frac{1}{2}\left( {1 - \frac{{\left| {\mathop \int \nolimits_{0}^{T} \tau_{wall} dt} \right|}}{{\mathop \int \nolimits_{0}^{T} \left| {\tau_{wall} } \right|dt}}} \right)$$

#### Endothelial cell activation potential (ECAP)

The endothelial cell activation potential (ECAP) is an important metric proposed by Achille et al.^56^ for representing the zone with high OSI and low TAWSS. High ECAP value indicates the endothelial susceptibility^40^ and can be calculated as,

20 $${\text{ECAP }} = \frac{OSI}{{TAWSS}}$$

## Results

### Validation

**Figure 3:**
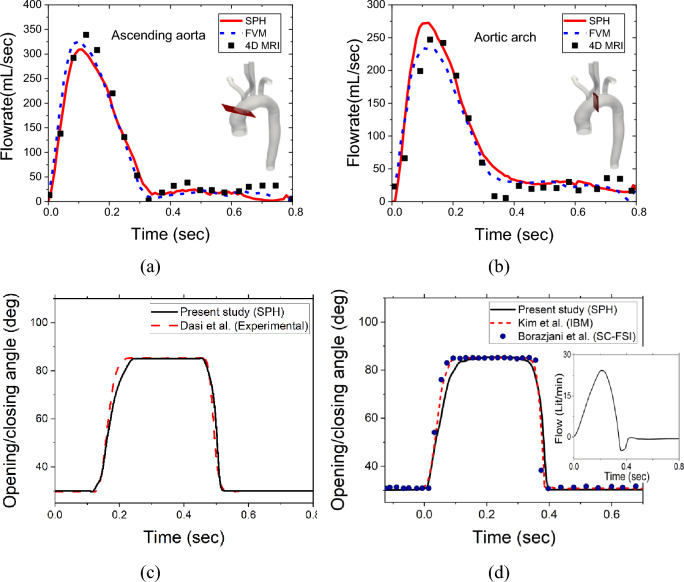
(**a**,**b**) Comparison of the flow rate from the present simulation with the FVM and 4D MRI data^40^; (**c**,**d**) Comparison of the SPH results with the experimental and IBM studies^13,58,59^.

The results obtained from the developed computational model have been validated against different set of data including FVM simulations, clinical data, and ex-vivo experiments. To validate the model of the patient specific geometry, flowrate from the present simulation has been compared against the flowrate obtained by the FVM simulations as well as 4D MRI dataset (without any valves), conducted by Deyranlou et al.^40^. As shown in Fig. 3a,b, the results from the patient specific SPH simulation exhibit an excellent agreement with the 4D MRI data and FVM simulation at two different locations along the aorta, building further confidence in the present approach.

It is worth noting that it would be difficult to validate the movement of the valves because of the patient specific vasculature. The valves exhibit a particular opening/closing behaviour according to the specific aortic geometry. Therefore, the movement of the bi-leaflet mechanical heart valve fitted in a straight tube aorta has been validated against the experimental study of Dasi et al.^13^. The leaflet starts to open at 0.15 s from the fully closed condition (30°) and reaches its maximum opening angle of 85° within 0.25 s. After maintaining the 85° position for another 0.25 s, the rapid closure of the valve starts before 0.5 s. As shown in Fig. 3c, SPH simulations typically indicate a 23 ms delay in valve opening compared to experimental observations, resulting in a maximum underprediction of 5.6% during the entire valve opening phase (spanning from t = 0.11 s to t = 0.52 s). In the SPH method, particles require a certain amount of time to settle around the geometries they come into contact with, leading to a minor time offset in the plots. Consequently, minor deviations or offsets from experimental observations are common in SPH and have been reported previously^24,57^.

From a clinical perspective, the observed delay in valve opening by SPH remains significant, highlighting a limitation in the current SPH methodology that warrants further investigation. Despite this discrepancy, there is generally strong agreement between SPH and experimental data from Dasi et al.^13^ regarding the overall opening/closing profile. This suggests the potential applicability of the current methodology for such purposes, with the need for continued refinement and exploration.

Furthermore, the opening/closing behaviour of the leaflet from the present model has been compared with the simulation results of Borazjani et al.^58^ (curvilinear immersed boundary strong coupling FSI) and Kim et al.^59^ (immersed boundary) as shown in Fig. 3 (d). The present simulations display good agreement with the results of Borazjani et al.^58^ and Kim et al.^59^, albeit a negligible deviation can be observed before reaching the maximum position. It is noted that for the 2nd and 3rd comparison of the leaflet dynamics, different inlet profiles have been chosen, as shown in Fig. 3d inset.

The current study introduces a novel approach for evaluating wall shear stress from SPH perspective, the details of which are provided in supplementary document. To validate the model, the estimated WSS values obtained from the proposed approach were compared with the analytical results by simulating a simple Poiseuille flow with a known velocity profile, and the WSS was estimated at the last particle layer at the wall boundary. As shown in the supplementary document, the WSS values obtained from the SPH simulation matched reasonably with the analytical results during transient conditions. In addition, the WSS was recalculated under turbulent conditions (Re = 9000) using the same geometry, and the results were compared to LES simulations obtained by ANSYS-Fluent (V2022.R1). Although the turbulence model slightly overpredicts the FVM results, the discrepancy was within an acceptable range.

### Hydrodynamic metrics

In the present work, a physiologically accurate pulsatile velocity profile has been imposed at the inlet (see Fig. 1b). As shown in Fig. 1c, the model studied here has five outlets located at the right subclavian artery (1), right common carotid artery (2), left common carotid artery (3), left subclavian artery (4), and the descending aorta (5).

**Figure 4:**
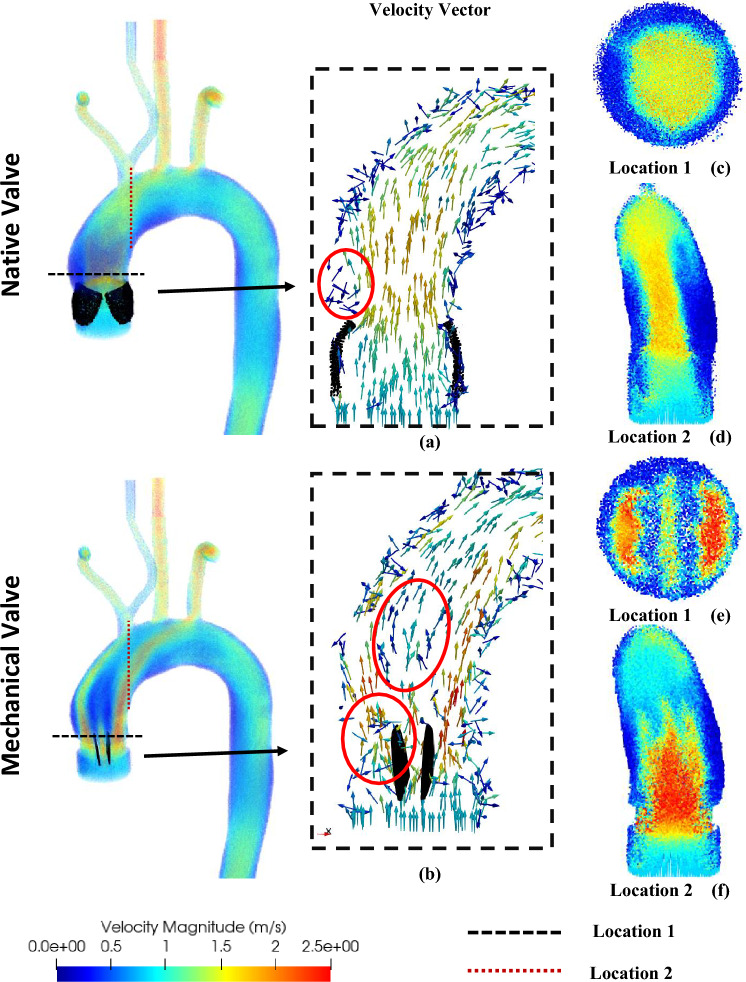
Velocity contours and velocity vectors at peak systole in the aorta with (**a**) the native and (**b**) the mechanical heart valve. Velocity contours at valve region and aortic arch regions for (**c**,**d**) the native and (**e**,**f**) the mechanical heart valves.

As discussed earlier, the present work considered two different cases. It may be note that the native heart valve has three separate tissue leaflets, while the mechanical aortic valve has two rigid hinged leaflets. Figure 4 shows that during the peak systole condition, the native valve opens completely, resulting in a distinct blood flow jet. This is followed by a small recirculation zone (red-circled) at the left side of the valve, attributed to a sudden enlargement of this specific aortic geometry.

However, under the same scenario, the bi-leaflet mechanical heart valve produces three separate blood flow jets, including one central and two lateral jets. Additionally, it is observed that the blood velocity increases with the mechanical valve and may reach as high as 2.5 m/s during the peak systole. The right lateral jet has the highest velocity magnitude due to this particular aortic geometry. The non-uniform distribution of the velocity profile results in a chaotic velocity vector behind the left leaflet and introduces velocity disturbances downstream of the valve, as highlighted in red circles. The bi-leaflet mechanical heart valve exhibits a lower effective orifice area (EOA) compared to that of the native valve, as shown in Fig. 4c,e. The reduced EOA, consequently leads to a higher pressure drop, which is certainly physiologically undesirable^60^.

**Figure 5:**
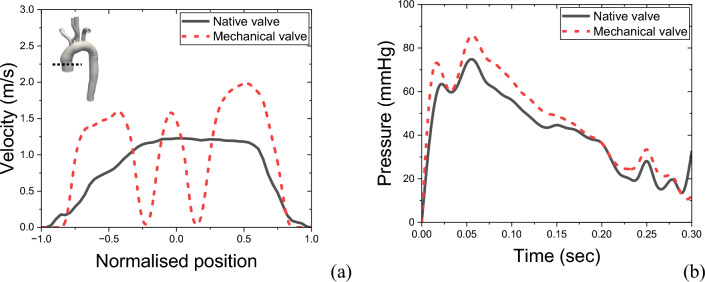
(**a**) Velocity profile and (**b**) surface average pressure variation at the inlet for the native and mechanical valves.

In order to better understand the flow pattern, the blood flow velocity profile has been taken at the vicinity of the valve and shown in Fig. 5a. It can be observed that unlike the native heart valve, the mechanical valve exhibits three different jets, as discussed in conjunction with Fig. 4. The asymmetrical nature of the lateral jet on either side of the leaflets is evident, with the left jet having a lower velocity maxima. This is associated with the asymmetric opening of the leaflets of the valve due to the aortic structure around the aortic root.

When assessing heart valves, it is important to strive for a lower pressure drop and a lower opening pressure. Figure 5b reveals that the mechanical heart valve has an opening pressure that is at least 15 mmHg higher than that of the native valve, increasing the afterload and requiring the left ventricle to work harder. Additionally, it is noteworthy that during peak systole, the maximum pressure drop for the bi-leaflet mechanical heart valve is 17.64 mmHg, whereas the native valve only induces a maximum pressure drop of 9.27 mmHg.

### Hemodynamic metrics

The magnitude of the WSS depends on the velocity of the blood flow, the viscosity of the blood, and the vessel geometry. The peak systolic wall shear stress (PSWSS) typically falls within the normal range of 5 Pa^61^, and exceeding this limit may cause damage to endothelial cells. This damage can initiate a series of events that ultimately result in the development of atherosclerotic plaques^3,62,63^.

**Figure 6:**
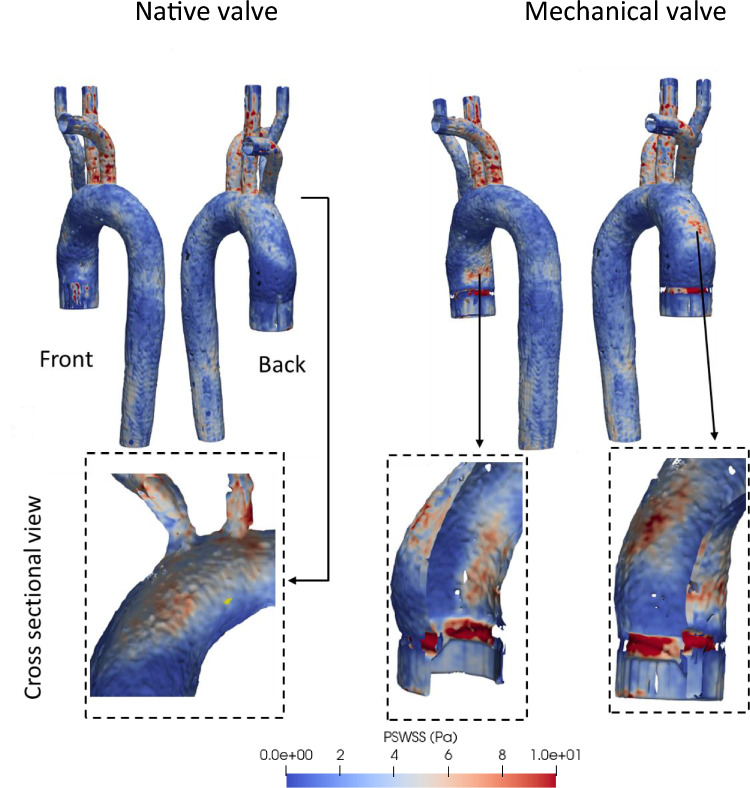
PSWSS variation across the aorta for the native and mechanical valves.

Variation of the PSWSS (at t = 0.1 s) has been illustrated in Fig. 6 with the cross-sectional view of the aorta fitted with both native and mechanical valves. Elevated PSWSS emerges as a direct catalyst for cellular damage, leading to the explosion of thrombus growth with uncontrolled platelet aggregation^64^. Generally, heightened PSWSS levels manifest particularly in the subclavian and carotid arteries, as well as the aortic arch (the point of branching), both in cases of natural and artificial valves.

Remarkably, in the case of mechanical valves, additional regions along the anterior and posterior side of the ascending aorta reveal PSWSS levels surpassing 8 Pa. It may be noted that the mounting ring is found to have the most elevated PSWSS readings (nearly 10 Pa at peak systole), which is a clear thrombogenic zone arising due to the MHV.

**Figure 7:**
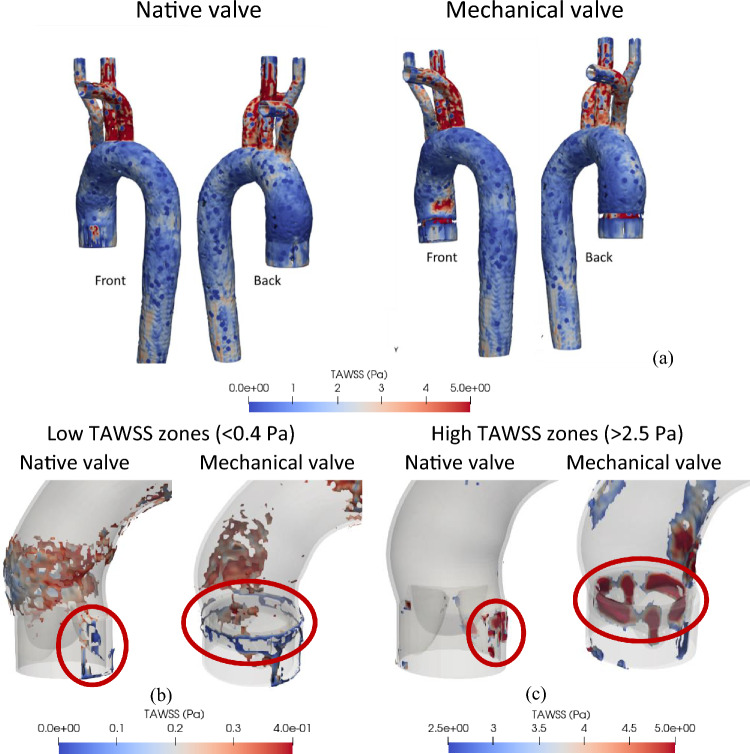
(**a**) TAWSS variation across the aorta for native and mechanical valves; (**b**) areas with low TAWSS; (**c**) areas experiencing high TAWSS.

It is worth highlighting that due to the pulsatile flow behaviour, TAWSS would represent a more meaningful biomarker of the endothelial cell damage compared to PSWSS. Therefore, Fig. 7a shows the TAWSS variation, where in most parts of the aorta, the values are within a standard physiological range of the TAWSS which can be considered to be between 0.4–2.5 Pa^40^. For both native and mechanical valves, the subclavian and carotid arteries exhibit much higher TAWSS levels^40^. In the case of the native valve, the aortic wall near the valve’s leaflet exhibits higher TAWSS values (marked in red), while a low TAWSS zone is observed near the valve region (marked in red) and at the ascending aorta (Fig. 7b). In the case of the aortic arch, the curvature of the vessel generates complex flow patterns that can result in high wall shear regions near the inner wall of the arch.

The presence of a mechanical valve exacerbates this effect, as the valve housing can create regions of flow disturbance that further increase the TAWSS. The inner mounting area of the mechanical valve is particularly susceptible to high TAWSS as illustrated by red circles in Fig. 7c. It can be observed that for the native valve case the ascending aorta exhibits a low TAWSS zone ranging between 0.2–0.4 Pa. On the other hand, for the case of mechanical valve the posterior side of the ascending aorta and aortic arch a experience low TAWSS levels followed by a very low TAWSS (< 0.2 Pa) region present at the outer surface of the valve mounting zone.

**Figure 8:**
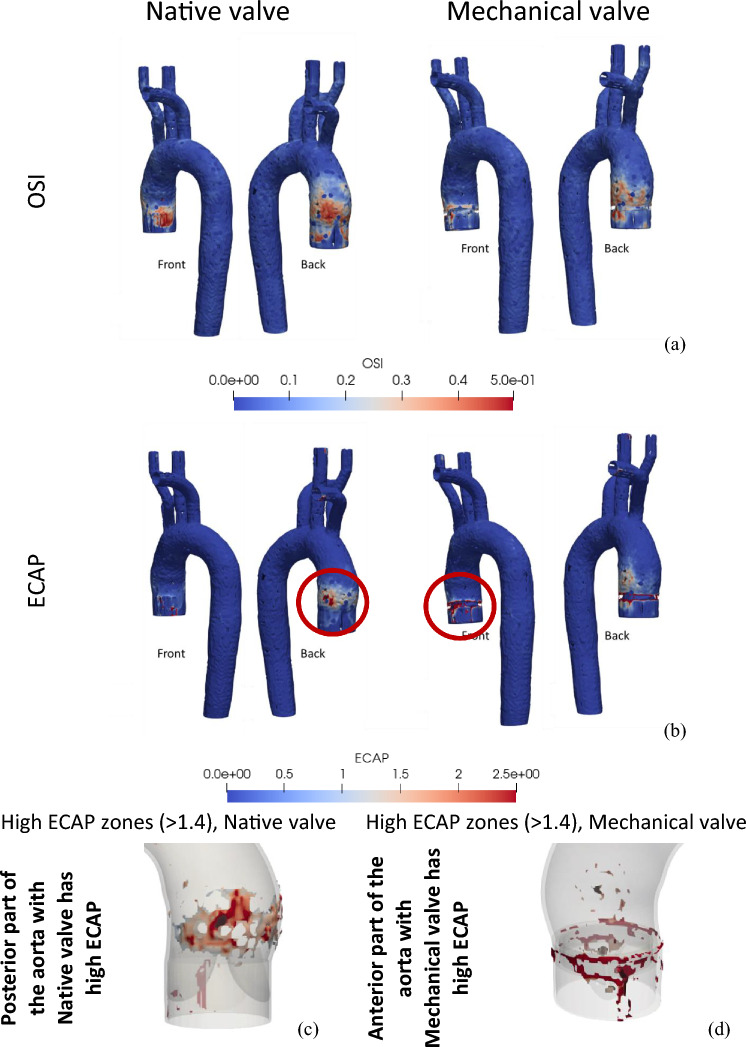
OSI (**a**) and ECAP (**b**) variation across the aorta for native and mechanical valves. The zones with high ECAP (**c**,**d**).

As mentioned earlier, OSI and ECAP are important metrics to interpret the effect of wall shear. Figure 8 illustrates the fluctuation of OSI and ECAP for both native and mechanical heart valves. OSI value of 0.5 generally corresponds to a 180° alteration in the direction of wall shear stress. In certain cases, high OSI values (> 0.25) can be correlated with lower wall shear stress and in fact, the results presented in Fig. 8a support this.

It can be observed from Fig. 8a that for both mechanical and native heart valves, the variation of the OSI is very similar. As one would expect, the zone near the valve and ascending aorta is more susceptible to high OSI levels. ECAP which indicated the zone of high OSI and low TAWSS has been depicted in Fig. 8b. It can be observed that a small region within the ascending aorta, near the valves, shows elevated ECAP. In addition, the edge of the mechanical valve mount exhibits substantially higher ECAP value (Fig. 8c,d, marked with red circles) which will be discussed further in the discussion section below.

**Figure 9:**
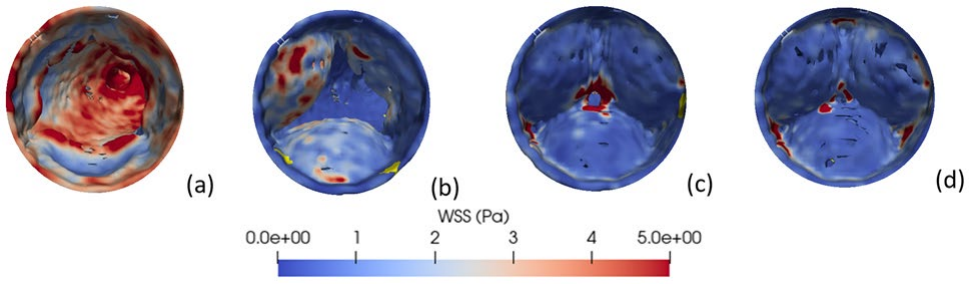
Closing behaviour and the WSS contours of the native valve at different time instance. from the ventricular side.

Figure 9 illustrates the peak systolic wall shear stress behaviour of the native valve's leaflet viewed from the ventricular side. Notably, during peak systole or when the valve is fully open, the leaflet edges experience elevated WSS due to the increased blood velocity. Subsequently, as the closing process commences, an asymmetrical leaflet movement is visible, likely influenced by the patient specific aortic structure. Figure 9b demonstrates that the leaflets closing first, experience higher WSS at their posterior surfaces (ventricular side). As the leaflets approach the verge of complete closure (as depicted in Fig. 9c), the untouched portion of the leaflets still displays a significant WSS level, which gradually diminishes as the valve closes entirely.

### Effective orifice area (EOA)

**Figure 10:**
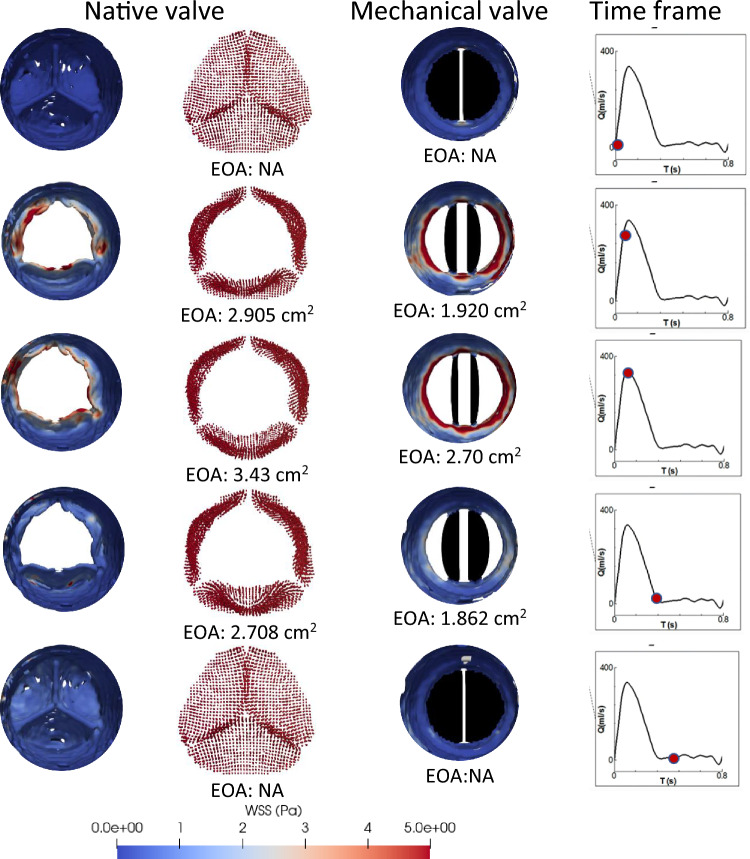
EOA variation of the native and mechanical valves at five different time frames.

EOA represents the area through which blood flows across the aortic valve during each cardiac cycle. Details of opening/closing behaviour as well as a comparison of the EOA for both valves, at different time frames over a cardiac cycle, has been shown in Fig. 10. It can be observed that the mechanical valve has lower EOA in all time frames compared to the native valve. For instance, at the peak systole phase, while the native valve has EOA of 3.43 cm^2^, the mechanical valve exhibits 21% lower EOA (i.e., 2.7 cm^2^).

The same trend is followed under acceleration and deceleration phases of the cardiac cycle. Lower EOA represents higher velocity and pressure drop, which are again physiologically undesirable. It is interesting to note that, at the acceleration and deceleration phases, the native valve shows marginal asymmetric movement in one of the leaflets (assigned as ‘leaflet 1’) due to the particular vascular structure.

Sudden enlargement behind leaflet 1 induces a low velocity/high pressure zone which restrict this leaflet in synchronisation with the other 2 leaflets. Moreover, the tip of the native heart valve from the aortic side shows a higher WSS compared to other sections of the valve, under the acceleration and peak velocity phase. However, at the deceleration stage, the outer surface of ‘leaflet 1’ (near the junction of this leaflet with the aortic wall) experiences high WSS value of approximately 5 Pa.

It is important to note that the contour plots depict iso-surface representations of the SPH particle dataset. Occasionally, due to complex aortic structure, these representations may contain minor holes in the surfaces (such as the aortic wall and leaflet) due to the approximation involved in the iso-surface conversion process from particle.

## Discussion

The impact of blood jet impingement on the surface of the aorta can be observed in Fig. 4c–f. In the case of the native valve, the jet hits the upper part of the aortic arch near the left common carotid artery. However, in the case of the mechanical valve, the central jet dissipates in the aortic arch and merges with the lateral jets. The left lateral jet flows directly towards the right subclavian outlet, while the right lateral jet merges with the left subclavian outlet. Figure 4d,f demonstrate that the flow pattern induced by the mechanical heart valve results in a non-uniform velocity field at the aortic arch. The merging of the lateral and central jets disrupts the natural haemodynamic of the blood flow, giving rise to local vortices. This is likely to increase the possibility of haemolysis and thrombosis^65–68^.

As was alluded to earlier, regions subject to significantly lower or higher WSS levels are likely to be more susceptible to thrombosis. The inner area of the mechanical valve mount exhibited a higher TAWSS levels, since the flow passage narrows, and the blood flow accelerates through the valve orifice. This is likely to cause damage to the endothelial cells, stimulating progression of the atherosclerotic lipid core^69,70^.

High WSS can also be a potential factor for developing high risk plaques. In the case of a mechanical heart valve, while the zone affected by high shear stress near the aortic arch may be small, the intensity of the TAWSS in that region is high. Elevated WSS can be associated with the enhancements in dense calcium, as well as a decline in fibrous and fibrofatty tissue. This could potentially indicate a zone with a more susceptible plaque phenotype^70^. Moreover, the higher TAWSS observed at the inner surface the valve mounting zone can induce necrotic core, which could block the flow passage^70^. Figure 7c showed the exact zone affected by high TAWSS (> 2.5 Pa).

On the contrary, a decrease in TAWSS values prolongs the residence time of blood in a specific region, thereby facilitating localised thrombus formation. The literature reveals that areas characterised by TAWSS below 0.36 Pa demonstrate an increasing propensity for monocyte adhesion to endothelial cells, potentially leading to thrombogenesis^40^. Furthermore, patients with lower TAWSS values (< 0.4 Pa) have a higher likelihood of experiencing aneurysm-related events^71^, and this range of TAWSS may also promote atherogenesis^53^. The outer surface of the mechanical valve mount shows a very low TAWSS (< 0.2 Pa), which potentially means the outer surface of the valve mount is susceptible to enhanced adhesion of the monocytes, in turn, leading to severe thrombus formation.

Moreover, a safe threshold for ECAP, which represents the ratio of OSI to TAWSS, can be considered as 1.38 (max OSI: 0.5/min TAWSS: 0.36). Higher ECAP values signify regions with a combination of lower TAWSS and higher OSI, indicating an enhanced propensity for thrombus formation. Figure 8c,d demonstrated that the mounting zone of the mechanical valve exhibits a high ECAP value due to elevated OSI and reduced TAWSS. Likewise, the ascending aorta of the native valve experiences a high ECAP value. Here it is interesting to note in the case of the natural valve, a specific leaflet corresponds to a zone that displays a notable susceptibility for elevated ECAP values. This intriguing phenomenon finds correlation with the asymmetric motion of leaflet 1 of the natural valve, attributed to the distinctive geometry of the aorta. This motion gives rise to mild flow recirculation, as depicted in Fig. 4a, resulting in reduced WSS at neighbouring aortic wall, consequently leading to heightened ECAP along the adjacent wall. In drawing from the aforementioned discourse, it is posited that ECAP holds potential as a comprehensive singular biomarker for the identification of thrombogenic zones, offering a distinct advantage over the separate utilization of TAWSS and OSI.

The apex of the leaflets of the native valve consistently encountered elevated levels of wall shear stress (WSS) throughout the acceleration and peak velocity phases, as highlighted in Figs. 9 and 10, persisting until the valve achieved complete closure. The examination of the closing behaviour of native heart valves elucidates a marked asymmetry in leaflet movement compared to mechanical valves, resulting in a non-uniform distribution of wall shear stress on the surfaces of the leaflets. In instances of valve malfunction or improper closure, a discernible small leakage passage is observed, leading to backflow or regurgitation. This regurgitant or leakage flow further intensifies wall shear stress at the leaflet edges. It is noteworthy that when the leaflets overlap or close completely, the absence of a distinct zone with significantly high wall shear stress is observed. Alongside as shown in Fig. 10, examining the cross-sectional view of the aorta near the mechanical valve mount reveals a clear depiction of the high wall shear along the periphery of the aorta. This implies that the inner surface of the aorta is expected to experience tearing due to the elevated shear forces. Subsequently, the mechanical valve may not achieve appropriate closing contact, leading to paravalvular regurgitation^72^.

For a better clinical representation, prediction of the mechanical valve induced thrombosis and affected zones have been provided in Table 1. It outlines the zones which are likely to encounter the associated clinical conditions including thrombosis, plaque formation, endothelial cell damage, thrombogenesis, atherogenesis, atherosclerotic lipid core, necrotic core formation, and valvular regurgitation. It highlights the importance of careful monitoring of WSS values in clinical settings, particularly in patients with mechanical heart valves that can alter blood flow patterns in the aorta. Accurate measurement and analysis of WSS can provide valuable insight into the haemodynamic factors that contribute to vascular pathologies and may help clinicians to develop effective preventive and therapeutic strategies.

**Table 1:**
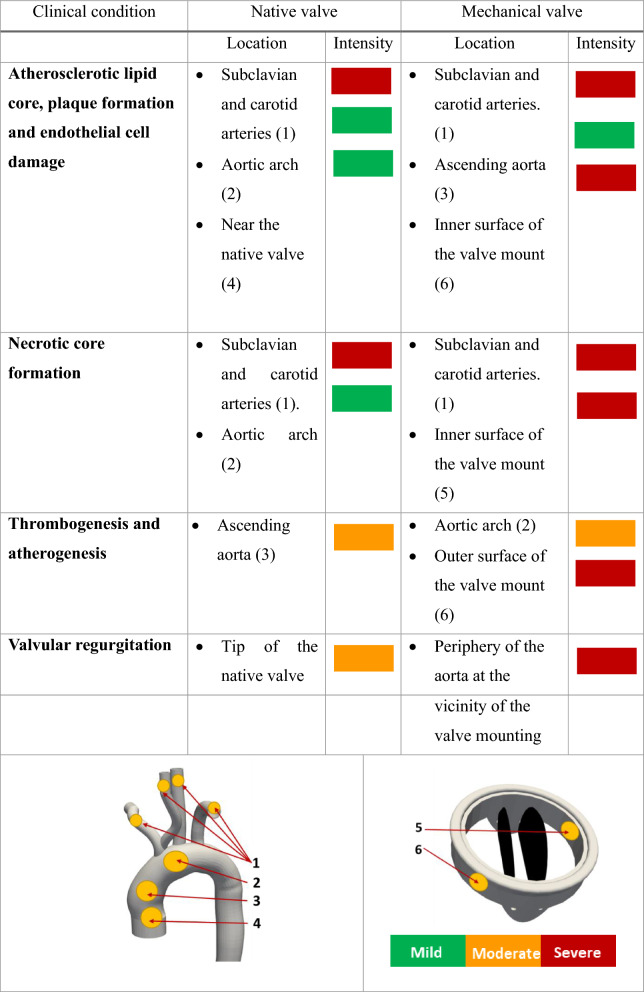
Various possible clinical complications and affected zones with severity.

From the above discussion, it is evident that by employing SPH, the implementation of the FSI techniques is simple as well as effective. The validation process, involving comparisons with ex-vivo experimentation and FVM studies, has demonstrated the remarkable accuracy of the current SPH results, exhibiting a deviation of less than 5%. Moreover, these results consistently align with established trends. The model proposed in this study excels in predicting potential hazards arising from the MHV. This predictive capability finds substantial support from various literary sources and real-world case studies. Unlike most conventional CFD methods used for cardiovascular flow modelling, in the SPH method, the dynamic mesh generation is not required, which in turn eliminates one of main challenges facing the in silico scientific community, arising from complex geometry and deformable boundary. Over the coming years, CFD and FSI are expected to emerge as essential tools for early detection of cardiac abnormalities and critical decision-making in clinical practice. However, in this study, the main focus has been given on a singular case study involving both native and mechanical valves, limiting our ability to perform statistical analysis on the presented results. Future work could involve applying the same model to a larger dataset of real case studies, allowing for a more comprehensive statistical analysis to enhance the scientific rigor of the findings. In addition, in future the scope of the present model can be enhanced by implementing the calculation of the residence time explicitly in the code. This SPH-FSI approach not only applies to valvular pathologies but also incorporates a wide range of cardiovascular intricacy. The effortless integration and remarkable efficacy achieved through the coupling of SPH with FSI methodologies are the main factors contributing to its success.

### Supplementary Information

Supplementary Information.

## Data Availability

All data generated or analysed during this study are included in this published article [and its supplementary information files].
