# Supplementary material for: Smoothed particle hydrodynamics based FSI simulation of the native and mechanical heart valves in a patient-specific aortic model
Source: Sci Rep. 2024 Mar 21;14:6762. doi: 10.1038/s41598-024-57177-w (PMC10957961; doi:10.1038/s41598-024-57177-w)
Supplement: Supplementary file 1 — Supplementary Information. [file 41598_2024_57177_MOESM1_ESM.docx]

Smoothed Particle Hydrodynamics Based FSI Simulation of the Native and Mechanical Heart Valves in a Patient-Specific Aortic Model

Sumanta Laha^1,2^, Georgios Fourtakas^1^, Prasanta K. Das^2^, Amir Keshmiri^1,3^

^1^ School of Engineering, University of Manchester, Manchester, M13 9PL, UK

^2^ Department of Mechanical Engineering, IIT Kharagpur, Kharagpur-721302, India

^3^ Manchester University NHS Foundation Trust, Manchester, M13 9PL, UK

**Supplementary document**

## Laminar viscosity and Sub-Particle Scale (SPS) turbulence

Instead of the widely used artificial viscosity scheme, a combination of Shao and Lo operator a large eddy Sub-Particle Scale (SPS) ^1^ turbulence model has been used in the present study as described by Dalrymple and Rogers ^2^ to define the dissipation term. In this scheme, the momentum equation is given by,

| $\frac{D\mathbf{u}}{\mathrm{Dt}}=-\frac{1}{\rho}\boldsymbol{\nabla}p+\mathbf{f}+\nu\nabla^{2}\mathbf{u}+ \frac{1}{\rho}\boldsymbol{\nabla}.\vec{\tau}$ | (S1) |
| --- | --- |

where $\nu\nabla^{2}u$ is the laminar viscous stress and it can be expressed as ^3^,

| $\left( \nu\nabla^{2}\mathbf{u} \right)_{i}= \sum_{j} m_{j}\left( \frac{4\nu r_{ij}\cdot\boldsymbol{\nabla}_{i}W_{ij}}{\left( \rho_{i}+\rho_{j} \right)+(r_{ij}^{2}+ 0.01h^{2})} \right)\mathbf{u}_{ij}$ | (S2) |
| --- | --- |

The last term in the right-hand side of Eq. (8) is accounted for by the SPS stress tensor and that term can be expressed by the means of Favre averaging which is generally used to compute compressibility in weakly compressible SPH ^2^. The term is as follows,

| $\left( \frac{1}{\rho}\boldsymbol{\nabla}\cdot\tau\right)_{i}=\sum_{j} m_{j}\left( \frac{\tau_{i}+\tau_{j}}{\rho_{i}\rho_{i}} \right)\cdot\left( \boldsymbol{\nabla}_{i}W_{ij} \right)$ | (S3) |
| --- | --- |

here, $\tau$ is the SPS stress tensor which has been calculated from the velocity gradient tensor and the eddy viscosity. The calculation of the $\tau$ has been explained in the WSS section of the main manuscript (Eq 11-12).

Therefore, the momentum mentioned in the Eq. (S1) can be expressed in SPH terms by,

| $\frac{\mathbf{Du}}{\mathbf{D}\boldsymbol{t}}\mathbf{=-}\sum_{\boldsymbol{j}} \boldsymbol{m}_{\boldsymbol{j}}\left( \frac{\boldsymbol{p}_{\boldsymbol{i}}\boldsymbol{+}\boldsymbol{p}_{\boldsymbol{j}}}{\boldsymbol{\rho}_{\boldsymbol{i}}\boldsymbol{\rho}_{\boldsymbol{i}}} \right)\left( {\boldsymbol{\nabla}_{\boldsymbol{i}}\boldsymbol{W}}_{\boldsymbol{ij}} \right)\boldsymbol{+}\mathbf{f}\boldsymbol{+}\sum_{\boldsymbol{j}} \boldsymbol{m}_{\boldsymbol{j}}\left( \frac{\boldsymbol{4}\boldsymbol{\nu}\mathbf{r}_{\boldsymbol{ij}}\boldsymbol{\cdot}\boldsymbol{\nabla}_{\boldsymbol{i}}\boldsymbol{W}_{\boldsymbol{ij}}}{\left( \boldsymbol{\rho}_{\boldsymbol{i}}\boldsymbol{+}\boldsymbol{\rho}_{\boldsymbol{j}} \right)\boldsymbol{+(}\boldsymbol{r}_{\boldsymbol{ij}}^{\boldsymbol{2}}\boldsymbol{+ 0.01}\boldsymbol{h}^{\boldsymbol{2}}\boldsymbol{)}} \right)\mathbf{u}_{\mathbf{ij}}\boldsymbol{+}\sum_{\boldsymbol{j}} \left( \frac{\boldsymbol{\tau}_{\boldsymbol{i}}\boldsymbol{+}\boldsymbol{\tau}_{\boldsymbol{j}}}{\boldsymbol{\rho}_{\boldsymbol{i}}\boldsymbol{\rho}_{\boldsymbol{i}}} \right)\boldsymbol{\cdot}\left( \boldsymbol{\nabla}_{\boldsymbol{i}}\boldsymbol{W}_{\boldsymbol{ij}} \right)\boldsymbol{,}$ | (S4) |
| --- | --- |

## Equation of State

As the fluid is weakly compressible (i.e., WCSPH methodology ^4^),fluid pressure calculation is dependent on particle density through the use of an equation of state (EOS). The compressibility of the system is modified to artificially reduce the speed of sound. This allows for a reasonable time step reducing the computational cost. This restriction helps to keep density variations within a threshold of less than 1%, preventing significant deviations from an incompressible approach, Tait’s EOS is used ^5,6^ which is given by,

| $P= \frac{c^{2}\rho_{0}}{\gamma} \left( \left( \frac{\rho}{\rho_{0}} \right)^{\gamma}-1 \right)$ | (S5) |
| --- | --- |

where $\gamma=7$ is the polytrophic index, $\rho_{0}$ is the density of the reference fluid at the beginning of the simulation, and the $c$ is the speed of sound at the reference density. In the present case, *c* is taken as 40m/s unless otherwise stated.

## Time-step

In the present work, the Symplectic position Verlet time integration scheme ^7^ has been used. This scheme is time reversible and a second-order explicit scheme with a time accuracy of O(∆t^2^). Therefore, it is most suitable for Lagrangian modelling. Under the influence of viscous force, velocity Verlet half step is required to compute the desired velocity, acceleration, and density progression respectively. The scheme reads,

| $\mathbf{r}_{i}^{n+\frac{1}{2}}= \mathbf{r}_{i}^{n}+\frac{\Delta t}{2}\mathbf{u}_{i}^{n}$  $\mathbf{u}_{i}^{n+\frac{1}{2}}= \mathbf{u}_{i}^{n}+\frac{\Delta t}{2}\mathbf{F}_{i}^{n}$  $\mathbf{u}_{i}^{n+1}= \mathbf{u}_{i}^{n}+\Delta t\mathbf{F}_{i}^{n+\frac{1}{2}}$  $\mathbf{r}_{\boldsymbol{i}}^{\boldsymbol{n+1}}= \mathbf{r}_{i}^{n}+\Delta t\frac{\left( \mathbf{u}_{i}^{n+1}+\mathbf{u}_{i}^{n} \right)}{2}$ | (S6) |
| --- | --- |

where *n* is the current time step$.$ Next, for the density calculation at the half-time step of the symplectic position Verlet time integration scheme the following equations have been used.

|  | $\rho_{i}^{n+\frac{1}{2}}= \rho_{i}^{n}+\frac{\Delta t}{2}D_{i}^{n}$  $\rho_{i}^{n+1}= \rho_{i}^{n}\frac{2-\varepsilon_{i}^{n+\frac{1}{2}}}{2+\varepsilon_{i}^{n+\frac{1}{2}}}$  $\varepsilon_{i}^{n+\frac{1}{2}}=-\left( \frac{D_{i}^{n+\frac{1}{2}}}{\rho_{i}^{n+\frac{1}{2}}} \right)\Delta t$  $\frac{D\mathbf{u}_{i}}{\mathrm{Dt}}=F_{i}; \frac{D\rho_{i}}{\mathrm{Dt}}=D_{i}; \frac{D\mathbf{r}_{i}}{\mathrm{Dt}}=\mathbf{u}_{i}$ | (S7) |
| --- | --- | --- |

In order to avoid irregular particle distributions and to increase the accuracy of the interpolation, the shifting particle algorithm of Skillen et al ^8^ is used, which is a quasi-ALE approach.

**B. Estimation of Wall Shear Stress**

In the SPH method, at every time step, the position of the particles changes according to the velocity and pressure field, therefore, it is erroneous to calculate the cumulative effect of the WSS in a particular wall position. In order to address this issue, a new set of WSS has been calculated for the boundary particles (which do not move with the time) based on the existing WSS data of the neighbouring fluid particles. This enables accurate estimation of the WSS parameter on the wall (i.e., Boundary particle). In order to perform this, an SPH summation has been carried out for all boundary particles as given in Eq. (S8):

| ${WSS}_{i}=\sum_{j}^{N} WSS_{j} W_{ij} V_{j},$ | (S8) |
| --- | --- |

where, ${WSS}_{i}$ is the interpolating boundary particle for which WSS is calculated while ${WSS}_{j}$ is the neighbouring fluid particle, bearing previously calculated WSS value based on Eq. (19). $W_{ij}$is kernel and $V_{j}$ is the volume of the particles.

Nevertheless, when it comes to particles at the boundary, the kernel receives partial support from adjacent fluid particles, while on the other side it gets truncated or supported by neighbouring boundary particles that carry null WSS information. Consequently, this approach might lead to an underestimation of the predicted WSS values. To mitigate this error, a kernel correction technique has been implemented, based on the Shepard's interpolation method proposed by Bonet and Lok^9^. Here, the kernel is adjusted to guarantee the precise interpolation of polynomial functions up to a specified degree. Instead of a computationally expensive linear correction, the calculation is simplified by using constants. Therefore, the corrected WSS can be calculated by Eq. S9.

| ${WSS\_corrected}_{i}=\frac{\sum_{j}^{N} WSS_{j} W_{ij} V_{j},}{\sum_{j}^{N} W_{ij} V_{j},}$ | (S9) |
| --- | --- |

| (a) | (b) |
| --- | --- |
| Figure S1. Validation of the WSS model with laminar analytical result (a) and LES turbulent FVM results (b) in a straight tube. | |

In Fig. S1, a detailed comparison has been shown between the WSS values obtained from present (SPH) model and those calculated analytically as well as via the finite volume method (FVM) for turbulent flow. Notably, present SPH simulation yielded WSS values that closely aligned with the analytical results, particularly during transient phases as shown in Fig. S1(a). While it is worth mentioning that the turbulence model predicted WSS values slightly differ from the FVM results, this discrepancy remained within acceptable limits, as illustrated in Fig. S1(b). This observation underscores the successful implementation of proposed WSS model.

Reference.

1. Gotoh, H., Shibahara, T. & Sakai, T. Sub-particle-scale turbulence model for the MPS method - Lagrangian flow model for hydraulic engineering. *Comput. Fluid Dyn. J.* **9**, 339 – 347 (2001).

2. Dalrymple, R. A. & Rogers, B. D. Numerical modeling of water waves with the SPH method. *Coast. Eng.* **53**, 141–147 (2006).

3. Lo, E. Y. M. & Shao, S. Simulation of near-shore solitary wave mechanics by an incompressible SPH method. *Appl. Ocean Res.* **24**, 275–286 (2002).

4. Monaghan, J. J. Simulating Free Surface. *Journal of Computational Physics* vol. 110 399–406 (1994).

5. Batchelor, C. . & Batchelor, G. K. *An introduction to fluid dynamics*. (Cambridge university press, 2000).

6. Monaghan, J. J., Cas, R. A. F., Kos, A. M. & Hallworth, M. Gravity currents descending a ramp in a stratified tank. *J. Fluid Mech.* **379**, 39 – 70 (1999).

7. Domínguez, J. M. *et al.* DualSPHysics: from fluid dynamics to multiphysics problems. *Comput. Part. Mech.* **9**, 867–895 (2022).

8. Skillen, A., Lind, S., Stansby, P. K. & Rogers, B. D. Incompressible smoothed particle hydrodynamics (SPH) with reduced temporal noise and generalised Fickian smoothing applied to body-water slam and efficient wave-body interaction. *Comput. Methods Appl. Mech. Eng.* **265**, 163–173 (2013).

9. Bonet, J. & Lok, T. S. L. Variational and momentum preservation aspects of Smooth Particle Hydrodynamic formulations. *Comput. Methods Appl. Mech. Eng.* **180**, 97–115 (1999).
